# Caring for family members with chronic physical illness: A critical review of caregiver literature

**DOI:** 10.1186/1477-7525-2-50

**Published:** 2004-09-17

**Authors:** Jung-won Lim, Brad Zebrack

**Affiliations:** 1 School of Social Work, University of Southern California, 669 MRF Building, West 34^th ^Street, & 102W, Los Angeles, CA 90089-0411, USA

**Keywords:** Family caregivers, Family stress theory, Quality of life

## Abstract

This article reviews 19 studies (1987–2004) on quality of life for family caregivers helping those with chronic physical illness. Here we explore the concepts of and instruments used to measure caregivers' quality of life. We were particularly interested in understanding stress-related variables and documenting factors influencing quality of life based on family stress theory. Findings show that various positive and negative terms equated with quality of life were used to measure them. Results indicate that stress-related variables as possible predictors influencing caregivers' quality of life include: patient and caregiver characteristics, stressors, stress appraisal, stress coping methods, and social support. Our recommendations touch upon applying theory for intervention, developing measurement, making operable the concepts for measuring, and the need for longitudinal and comprehensive study.

## Background

Recent reforms in U.S. health care systems mean that individuals with long-term, complex health problems are being cared for at home by family members [1]. Specifically, changes in medical practice resulting in shorter impatient hospital stays and the search for outpatient substitutes such as home-based care have brought cost savings to both hospitals and consumers. A study reported that home-based care reduced the cost per patient treated by 44% overall compared with hospital-based treatment [2]. Despite such cost-effectiveness, this trend means that an increased financial, physical, and emotional responsibility falls upon family members who care for a person with chronic physical illness [3]. Now, more than 25 million Americans serve as family caregivers for that population. Their work, if it were part of the market economy, would have an economic value of nearly $257 billion in 2000, which is equal to 20 % of the total for all health-care expenditures [4]. For example, family caregivers are more frequently called upon to use daunting and complex equipment at home. They also deal with extensive coordination of care, including symptom management, disability, mobility, and dressings. In the face of these increasing challenges and responsibilities, caregivers often feel tired, isolated, and overwhelmed, because they lack support, training, information and a sympathetic ear. Furthermore, some family caregivers who are employed report missing work, taking personal days, and quitting or retiring early to provide care [5]. Thus, chronic illness affects not only the lives of those suffering from disease but also those of family members who care for them. Attending to the impacts of chronic illness on family members is important because the physical and emotional health of family caregivers has the potential to influence the health, welfare and successful rehabilitation of persons with such chronic illness [6].

Existing studies document how caring for chronically ill family members or significant others at home influences multiple aspects of caregivers' lives. These effects are physical, psychological and social and may include worsened physical health, impaired social and family life, and increased stress, anxiety and depression ([7-9]). Placing these conditions experienced by caregivers in the context of family stress theory and quality of life advances our understanding of caregivers' experiences by examining how multiple aspects of caregivers' lives – their *quality of life *– may be partly influenced by other existing environmental stressors, stress appraisal, coping methods and social support.

The importance of family stress theory in studying normative family transitions and adaptation to major life changes and illness is based on the central role that family strengths and capabilities play in understanding and explaining psychological and behavioral outcomes [10]. In family stress theory, the family is "viewed as encountering hardships and changes as an inevitable part of family life over the life cycle" [11]. Given that caring for a seriously ill family member arguably is (or is quickly becoming) an inevitable part of family life, current research has begun examining family stressors, stress appraisal, coping methods, and social support as they influence QOL outcomes, or as they attenuate the effects of other patient or caregiver characteristics or health-related variables on caregiver QOL ([12-15]).

Quality of life is a construct that encompasses health and functioning, socioeconomic status, psychological, emotional and spiritual aspects, and family [16]. Ferrell [17] confirmed the influence of such multidimensional aspects on the QOL for breast cancer survivors. Wyatt & Friedman [18] also identified concerns related to QOL in those with chronic physical illness, and suggested that considering multidimensional aspects of QOL for them is essential. Although some scholars have different points of view regarding the dimensions of QOL, most researchers generally agree that QOL is multidimensional, subjective, and relating to a state of physical, psychological, social, spiritual well-being [19]. However, QOL for caregivers includes more aspects such as burden and family functioning [20]. Further, some researchers use QOL together with life satisfaction, adaptation, health, and distress ([12,13,21-23]). Such comprehensive consideration has led to an awareness of QOL as a broader and more appropriate concept for determining how caregiving affects family members [1]. Therefore, it is meaningful to address QOL comprehensively in this paper. This work will also consider 1) the lack of consensus on concepts like stressors, stress appraisal, coping methods, and social support, and 2) inconsistent results regarding factors influencing caregivers' QOL.

To address the lack of review papers on this topic as well as inconsistent results among empirical studies, we undertook a thorough review of the literature. We were particularly interested in research that reported psychosocial or QOL outcomes and accounted for family stress variables as correlates or explanatory variables. The purpose of this paper is twofold: to review caregiver QOL impacts (loosely-defined) organized around family stress theory, and to discuss the implications of findings for future research (i.e., measurement, hypothesis testing, refinement of concepts and constructs).

## Methods

Only published peer reviewed research articles were included in this review. Several methods were applied in searching the literature. First, articles were limited to those published in English between January 1, 1987, and January 31, 2004. Second, participants were caregivers of patients over 21 years of age with chronic physical illnesses. Third, a study was excluded if patients were receiving hospice care. Fourth, a computer search was conducted in February 2004 to review the databases of MedLine and PubMed by using the following key words: caregivers, caregiving, chronic illness, quality of life, adjustment, life satisfaction, burden, distress, and family stress theory. The terms "health," "stress," and "coping" were also used for study retrieval. Databases also were searched for review articles published during the same time period. Additional sources for empirical reports included reference lists from published studies. Of the more than 220 articles identified, there were only 16 empirical studies based on family stress theory that focused on the caregivers of patients with chronic physical illness and measured the QOL of caregivers. Two additional articles were identified from searching review articles. Finally, one more article was found in the references of these 18 articles. Thus, a total of 19 articles are included in this review (Appendix 1 [See additional file]).

## Results

### Concepts and Instruments Used to Measure Caregiver Quality of Life

Reviewed studies used diverse outcomes and models to indicate caregivers' QOL. Psychosocial outcomes falling with varied dimensions of QOL and measured included adaptation, mental health, life satisfaction, stress, emotional distress, health, caregiver burden, and depression. Given the variety of terms equated with QOL, both positive and negative terms were used to measure the QOL. Three studies ([12,13,21]) used positive terms such as 'adaptation' and 'life satisfaction.' Seven studies ([15,22-27]) measured QOL using negative terms such as 'caregiving burden,' 'depression,' 'stress outcomes,' and 'emotional distress.' Other research used neutral terms including 'health,' 'mental health outcomes,' and 'quality of life.'

Of 19 reviewed articles, only one used a single measure of QOL *per se*: the Caregiver Quality of Life Index containing items related to physical, emotional, social, and financial wellbeing [28]. Eight papers reported use of a single instrument based either on a modified patient questionnaire or on other concepts (burden, general health, and mood status) indicating various QOL dimensions. Ten reported studies administered a composite measurement combining some concepts. Table 1 shows diverse outcomes and instruments used to measure caregivers' QOL.

**Table 1:** Concepts and measurements of quality of life

**Outcome (QOL)**	**Single Measurement**	**Composite Measure**
Quality of life	1. Caregiver quality of life index – cancer [28]	1.1) Stress, 2) Anxiety, 3) Depression, 4) Health [7]
	2. Multidimensional quality of life scale – cancer [33]	2.1) Physical health, 2) Emotional health,
	3. Quality of life index [32]	3) Use of psychotropic drugs,
		4) Caregivers' social life,
		5) Financial status [30]

		1.1) Depression, 2) Life satisfaction, 3) Health [12]
Adaptational outcome (Adaptation)		2.1) Caregiver's level of life satisfaction,
		2) Depression
		3) Subjective symptoms of stress [13]

Mental health outcome		1.1) Depression, 2) Quality of life [29]

Life satisfaction	1. 8-item, open-ended questionnaire [21]	

Stress outcome (stress response, distress)	1. Brief symptom inventory [34]	1.1) Yielding of role, 2) Physical health, 3) Anxiety [24]
		2.1) Anxiety, 2) Depression, 3) Stress [25]
		3.1) Burden, 2) Depression, 3) Anxiety [26]

Emotional (Psychological) distress	1. Profile of mood states – short form [22]	
	2. General health questionnaire [23]	

Health	1. the Medical Outcome Study 36-item	1.1) Caregiver mental health,
	Short Form Health Survey [31]	2) Caregiver physical health [14]

Caregiving Burden	1. Zarit burden scale 27	

Depression		1.1) depression, 2) strain [15]

### Factors Influencing Caregiver Quality of Life

Studies of variables influencing caregivers' QOL are summarized in Table 2. Variables examined are categorized here into patient characteristics, caregiver characteristics, stressors, stress appraisal, stress coping methods, and social support.

**Table 2:** Factors influencing caregiver quality of life

**Predictors**		**Significant Mediating variables**	**Quality of life**	
			
			**Significant**	**Not found**
***Patient characteristics***				
Performance status			[7], [24], [26], [28], [34]	
Age			[7]	[15]
Gender			[15]	
Depression			[7], [29]	
The kind of illness			[30]	
Pain / symptoms (severity of illness)				[29, 32]

***Caregiver characteristics***				
Age			[7], [24]	[15]
Gender			[7], [15]	[24]
Physical disability			[7]	
Income			[29], [32]	
Initial quality of life			[29]	
Educational level			[22], [28]	
Health problem			[26]	
Depression			[29]	
Anger			[26]	
Anxiety			[26]	

***STRESSOR***				
***Primary***	Objective context		[13]	[14]
	Caregiving demands			[13], [22]
	Patient impairment		[12], [15]	
	Duration of care			[29], [25]
	Intensity of care			[29], [25]
	ADL Dependency		[7], [24], [26]	[29]
	Stress types		[25]	
	Caregiver overload		[24]	
	Recurrence		[15]	
	Problem behavior		[24]	

***Secondary***	Subjective context		[13]	
	Caregiving demands		[13]	
	Role change		[21]	[29]
	Responsibility		[21]	
	Caregiver experiences	[29]		
	Life style interference	[22]		

***Stress Appraisal***				
Appraisal		[12], [14]	[12], [14], [23], [31]	
Perceived control			[13]	
Differences in the perception (pt & caregiver)			[33]	

***Stress Coping Methods***				
Coping responses		[12], [13], [25], [14], [15]	[12], [14], [26], [31]	[24], [32]

***Social support***				
Perceived adequacy of social support		[14], [25], [34]	[14], [21], [26], [31]	
Social life and social network		[12], [15],	[12], [7]	
Family life (Quality of relationship and Marital adjustment)			[7], [29], [32]	
Loneliness			[7]	
Resources			[26]	
Formal support		[24]		

### Patient characteristics

Nine articles examined the association between patient characteristics (including performance status, age, gender, depression, type of illness, pain, and symptoms) and caregiver QOL. Generally, there were significant correlations between caregiver QOL and the patient's physical and emotional characteristics as related to the illness. Seven studies ([7,29,24,26,28,31]) found that the patient's performance status, type of illness, and depression were related to the caregiver's QOL. However, two ([29,32]) showed that pain and physical symptoms were not related to the caregiver QOL. Two other articles investigated the relationship between patient age and caregiver QOL, but there were no consistent results. Schumacher, Dodd, & Paul [15] reported a relationship between patient gender and caregiver QOL, with caregivers of male patients reporting higher levels of strain.

### Caregiver characteristics

Eight of 19 articles examined the relationship between caregiver characteristics and QOL. A caregiver's age, gender, physical disability, income, initial QOL, educational level, health problem, depression, anger, and anxiety were addressed as characteristics. Three studies examined the association between a caregiver's age and QOL, with two ([7,24]) reporting that older age of the caregiver is associated with increased stress. Two of three articles investigating the relationship between a caregiver's gender and QOL reported that females were more likely to be depressed ([7,15]). In addition, the caregiver's physical disability, income, initial quality of life, educational level, health problem, depression, anger, and anxiety were consistently found significant in reducing their QOL.

### Stressors

Eleven articles investigated the relationship between stressors and caregiver QOL, but they did not show consistent results. To varying degrees, caregiving demands, patient impairment, the duration and intensity of care, ADL (activities of daily living) dependency, stressor types, caregiver overload, how much assistance is given the caregiver, recurrence of illness and problem behavior in the patient were identified as primary stressors. Secondary stressors were caregiving demands, role change, responsibility, caregiver experience, and life-style interference. Seven studies ([7,12,13,15,24-26]) found that primary stressors were related to reductions in caregiver QOL. However, five articles ([13,14,22,29,25]) did not find any association between primary stressors and QOL. Two ([13,21]) found a significant relationship between secondary stressors and lower QOL. In contrast, another [29] found that secondary stressors were unrelated.

Vedhara, Shanks, Anderson, & Lightman [25] investigated the relative importance of stressor types on stress outcomes. Their study demonstrated that stressor types (e.g. daily hassles, caregiving-specific stressors, and life events) determined the stress outcomes, with the proportion of variance accounted for by the stressor indices (which ranged from 20% to 53%). Winslow [24] found that caregiver overload was positively related to caregiver anxiety. The hypothesized direct effect of a care receiver's problem behavior on the caregiver's yielding up his/her role was also supported by the findings. That is, higher levels of a care receiver's problem behavior as the primary stressor were more likely to lead to patient institutionalization. Examining the stress process in family caregivers of persons receiving chemotherapy, Schumacher et al. [15] corroborated that modest but significant negative relationships were found between caregiver strain and patient functional status as well as disease recurrence. Haley, Levine, Brown, & Bartolucci [12] also found a significant positive correlation between patient impairment on the IADL (instrumental activities of daily living) and caregiver depression scores. Wallhagen [13] found that the subjective context and subjective demands of caregiving as well as the objective context were associated with the caregiver's adaptation, including level of life satisfaction, depression, and subjective symptoms of stress. Aspects of the caregiving situation assessed by both the objective and subjective context indices included caregiver competence, social resources, the physical environment, and socioeconomic status or perceived financial adequacy. The objective and subjective demands of caregiving included caregiving responsibilities, instrumental activities of daily living, and personal demands.

Examining determinants of caregiver outcomes through a longitudinal study, Nijboer, Trienmstar, Tempelaar, Sanderman, & Van den [29] considered various stressors as both mediating and predicting variables. Some stressors, including duration of care, intensity of care, ADL dependency of patients, and role change, were not related to caregiver QOL. On the other hand, caregiver experience (i.e. disrupted schedule, financial problems, lack of family support, loss of physical strength, and self-esteem) was a significant mediating variable affecting the relationship between caregiver, patient, care characteristics and a caregiver's mental health. They found that all caregiver experiences were related in the expected direction to the caregiver's level of depression. With regard to a caregiver's QOL, only the loss of physical strength and its impact on the caregiver's self-esteem appeared to be related significantly, also in the expected direction. Negative caregiver experiences were associated with low income, living with the patient exclusively, distressed relationship, high level of patient dependency, and high involvement in caregiving tasks. Cameron, Franche, Cheung, & Stewart [22] specifically examined the mediation of lifestyle interference as the secondary stressor between the amount of care provided and emotional distress. The results supported that lifestyle interference mediated the relationship between caregiving assistance and overall mood disturbance. However, they did not find the relationship between caregiver QOL and caregiving demands to be the primary stressor.

### Coping methods

Eight reports examined the relationship between coping methods and caregiver QOL. Five studies ([12-15,25]) found that stress coping methods operated as a mediating variable affecting the relationship between predictor variables and caregiver QOL. Four studies ([9,12,14,26]) concluded that coping methods significantly predicted the caregiver's QOL. Two studies ([12,14]) showed that stress coping methods operated as both predictor and mediating variables, where two others ([32,24]) found no such significant relationship.

Haley et al. [12] found that coping responses were significant mediators and predictors of all three outcome variables: depression, life satisfaction, and self-related health. They showed that when caregivers used logical analysis and problem-solving coping strategies, they enjoyed a higher QOL. Information seeking was related to a better health outcome, and affective regulation was related to better outcomes in health and life satisfaction. Emotional discharge was actually related to higher levels of caregiver depression. Goode, Haley, Roth, & Ford [14] found that initially higher proportions of approach versus avoidance coping predicted better health over time. This suggests that relatively greater use of approach coping may help optimize caregivers' health over time. They also examined mediated effects on physical and mental health outcomes. Changes in approach coping percentage were directly related to changes in depression for self-care stressors and memory and behavior problems, indicating that as relative levels of approach coping increase, depression decreases.

Of the four coping strategies, Wallhagen [13] discovered that, only wishful thinking mediated the perceived control and outcome variables. That is, wishful thinking coping behavior had a negative relationship with all adaptation variables. The higher levels of perceived control also reported using wishful thinking behavior. Schumacher et al. [24] studied how coping mediated the relationship between strain and depression. A modest but significant negative relationship was found between caregiver strain and coping efficacy. Predictably, caregiver depression was also significantly related to coping efficacy. That is, caregivers who experienced less coping efficacy were more depressed.

### Stress appraisal

Six articles examined the association between stress appraisal and caregiver QOL. Two ([12,14]) demonstrated that stress appraisal operated as both a predictor and mediating variable. Four articles ([33,13,23,9]) investigated how stress appraisal was associated with caregiver QOL as a predictor variable only. Thus, most research investigating the relation between stress appraisal and caregiver QOL showed their significant relationship.

Haley et al. [12] found that measures of caregiver appraisal were consistently related to caregiver outcome and operated as mediator and predictor variables. When caregivers appraised patients' behavioral problems and disability as highly stressful and appraised themselves as lacking in self-efficacy, they experienced higher levels of caregiver depression. Goode et al. [14] found that changes in one domain of caregiving stress, memory and behavior problems produced changes in stressfulness appraisals as a mediating process. Changes in stressfulness appraisals were then positively associated with changes in depression and health symptoms. These results may suggest that the appraised stressfulness of memory and behavior problems mediates the relation between these problems and caregiver health outcomes.

In contrast, Wallhagen [13] did not validate the hypothesis that perceived control mediates the objective and subjective aspects of caregiving and caregiver adaptation. However, he found that perceived control had a direct relationship with life satisfaction and depression. Thus, perceived control was associated with a higher level of life satisfaction and lower levels of depression and subjective symptoms of stress. In addition to its direct relationships with the outcome variables, perceived control also had an indirect relationship with both life satisfaction and depression through its direct connection to wishful thinking.

Miaskowski, et al. [33] investigated whether differences in patients' and family caregivers' perceptions of pain experience influence patient and caregiver outcomes. In terms of the QOL measures, significant differences were found for psychological well-being, interpersonal well-being, nutrition and the total QOL score, with reporting lower scores for patients whose pain intensity scores were non-congruent with their family caregivers. These data suggest that non-congruence in the patient's and the caregiver's perceptions of the patient's pain may result in a poorer QOL score for the patient.

### Social support

A majority of the articles addressed social support as a mediator and predictor variable. Six articles ([12,14,15,24,25,34]) reported that social support mediated both predictor and outcome variables. Seven ([7,12,14,21,29,26,32]) showed a direct relationship between social support and caregiver QOL. Specific items of social support addressed here were perceived adequacy of social support, social life, social network, family life (quality of relationship and marital adjustment), loneliness, resources, and formal support.

Examining predictors of adaptational outcome among dementia caregivers, Haley et al. [12] found that social support and activity were significant predictor variables of caregiver outcome. They discovered that higher levels of social network size, activity, and satisfaction with network were related to better outcomes, particularly life satisfaction and health. Social support and activity also mediated the stressors and caregiver outcomes, including depression, life satisfaction, and health. In the study conducted by Schumacher et al. [15], caregiver depression was significantly related to perceived adequacy of social support. Predictably, caregivers with less social support were more depressed. Social support was found to mediate the relationship between functional status and depression. Goode et al. [14] found that initial levels of social support also protected physical health changes over time. Those caregivers who reported higher initial levels of social support resources actually showed improved health over time. Initial satisfaction with level of social support provided the same beneficial effect in preventing physical health problems or promoting health improvements. Vedhara et al. [25] assessed the predictive stability of psychosocial mediators over a six month period. The results of the regression analyses revealed stable predictive relationships between the mediator factors and the stress response indices. Anxiety was predicted by seeking social support at six months. Ergh, Rapport, Coleman, & Hanks [34] also examined predictors of caregiver distress among 60 caregivers of patients with a traumatic brain injury. In this study, social support showed a direct relationship to family functioning. As well, social support powerfully moderated the caregiver's psychological distress. That is, in the absence of adequate social support, caregiver distress increased with performance status in care recipients. On the other hand, in studying how formal supports affect stress outcomes in family caregivers of Alzheimer's patients, Winslow [24] considered formal supports as mediators. He found that no formal support mediated primary stressors and caregiver characteristics in the directions hypothesized. Others ([21,26,7,29,32]) showed that social support was a predictor variable affecting caregiver QOL.

## Discussion

Nineteen studies have been reviewed to understand stress-related variables and to examine how each factor influences a caregiver's QOL. Factors were identified from the literature based on family stress theory and included patient characteristics, caregiver characteristics, stressors, stress appraisal, coping methods, and social support. In this section, we will discuss method as well as family stress theory based on the literature reviewed.

### Theoretical and Clinical Considerations

Family stress theory provides a way of viewing the family's efforts over time to adapt to multiple stressors through using family resources and perceptual factors as a coping process aimed at achieving family balance [35]. A family situation addresses multiple changes and demands simultaneously, not single stressors. Secondary stressors, such as role change, responsibility, and caregiving demands, emerge from the primary stressors and these strains often may be difficult to resolve. They become instead a source of chronic strain. Chronic strain causes a build-up of unresolved stressors and contributes to undesirable characteristics in the family environment [36]. Although most of the reviewed papers addressed stressors as factors influencing caregiver QOL, the focus was on primary rather than secondary stressors. As a result, researchers may be overlooking stressors without considering chronic illness as a source of chronic strain changing the family system. Therefore, it is imperative to consider secondary as well as primary factors for understanding stress on caregivers of patients with chronic physical illnesses.

Resources for the family are the psychological, social, interpersonal, and material characteristics of individual family members, of the family unit, and of the community. That meets family demands and needs. When families have insufficient resources, their needs and demands are not adequately met. As a result, this contributes to increased conflict in the family environment [35]. Although this theory emphasizes resources, the focus on resources is remarkably broad [26]. For instance, some works ([13,14,23,26]) have addressed social support and coping as components of resources. A lack of clarity is likely to bring inconsistent research results and thus may cause problems of generalization. Instead we need to clearly prescribe each concept.

Family stress theory suggests that stress may be perceived or experienced both as a crisis and a challenge to be overcome [10]. The family may perceive the stressor as having caused a crisis. Or the family may accept it and see it as a challenge. Perception of the stressor as a challenge suggests that, over time, families engaged in a constructive effort to manage the stressor will redefine their total situation [35]. This explanation regarding stress perception may artificially dichotomize the stressor as a crisis or a challenge. However, perception is so subjective that each family member may differently interpret an event occurring within a family system.

The subjective characteristics of perception may impede measuring the cognitive patterns of each person. Even if caregivers perceive stressors as a challenge and have volition to cope with stressors, we must consider the possibility that recovery from crisis may not occur among caregivers of patients with chronic illness. In other words, many caregivers may have financial burdens caused by continuous treatment and tests. Additionally, although personal characteristics change, environmental characteristics of patients and caregivers, such as social prejudice against the chronically ill, may not change. As shown by inconsistent results from previous research findings, the assumption that caregivers recover from the crisis and achieve some level of adaptation needs to be re-considered.

Family stress theory also emphasizes the need for intervention following clear assessment. In the Resiliency Model [37], practitioner interventions were directed at restoring the balance between family stressors and resources. This can be the first intervention to assess whether family behavior is adaptive or maladaptive. Robinson [38] maintains that in collaboration with the family it is necessary to develop a plan, for managing stress. The plan includes the following points: (a) commitment of all family members to work on the problems; (b) inclusion of all past successful coping strategies; (c) brainstorming of all possible strategies; (d) use of strategies that are flexible, reality-oriented, and open to expression of emotions; and (e) discussion of possible outcomes of all strategies. Figley & McCubbin [36] suggested that a family crisis should be an opportunity for family interventionists to promote family well-being. Social workers can not only make use of the community and its programs and services in support of families under stress, but also more importantly use the situation to improve the family's problem-solving skills, coping repertoire, and overall interpersonal relationships. Besides those interventions mentioned above, the reviewed papers suggested other interventions for caregivers and health care professionals on the basis of family stress theory. These interventions emphasized the need for health professionals' involvement in the ongoing care of cancer patients and their families to monitor increasing demands [29], the importance of educating family members in effective ways to communicate [33], and the need for long-term counseling and early involvement of caregivers [6].

Based on strategies mentioned above, social workers can use individual therapy, family therapy, education, and problem-solving programs as interventions for caregivers of patients with chronic illness. When social workers so apply family stress theory, they must maximize resources because resources can positively influence coping, perception, and adaptation. After finding resources, it may be helpful to educate caregivers, focusing on their perceptions, problems-solving, and coping skills. In family stress theory, resources, perception, and coping mutually interact, and thus may influence QOL among family members. As a result, synergistic interventions that integrate all factors may be more effective. Finally, family interventions must consider longer-term effects or the sustained effect, because coping methods and perception may change over time, depending on circumstances.

### Methodological Considerations

Some studies of caregivers and family stress theory address various outcome variables, including QOL, adaptation, life satisfaction, emotional distress, and caregiving burden ([12,13,28]). As well, most research uses concepts such as stress, perception, coping, and social support as predictor or mediating variables. These concepts are so subjective that they are hard to define and measure using concrete methods. For example, Schumacher et al [15]'s study measured both perceived efficacy of coping strategies and perceived adequacy of social support with only one-item indicators. Thus, at first, operationalization of concepts is required to reduce the gap between theory and research and clear the way for measure. As well, specific instruments to measure the complex and multidimensional phenomena need to be developed.

For a number of reasons, we need longitudinal studies in research applying family stress theory to caregivers of the chronically ill. Otherwise we cannot understand longer-term or sustained effects. First, although a patient with chronic illness may have completed treatment, recurrence of the illness is possible. Second, coping methods and appraisal may change depending on the circumstances, because family stress theory is dynamic and influenced by both internal and external environments. A longitudinal study may see the attrition of participants over time. Due to the nature of chronic illness, many patients may have died or experienced recurrence at the follow-up evaluation. Therefore, caregivers of these patients need to be followed through the end to assess predictors and consequences of patient death and recurrence of illness.

Family stress theory is very complex and comprehensive. As a result, it is difficult to conduct research using the full model. Examples of this complexity include interactions among various factors, interactions and transactions over time, individual dynamics within the family system, and the balance between family demands and the family. Considering these multidimensional aspects, it would take much hard work to test the full model of family stress theory. However, we need to try. Otherwise, we may never understand the complex relation between individual, family, and environment.

## Supplementary Material

Additional File 1 **Appendix 1: Review of empirical findings on quality of life of family caregivers. **Author, topic, significant predictor variables, mediating variables, outcome variables, measurement, and intervention of 19 reviewed articles are shown in detail. Click here for file
