# Supplementary material for: Caring for family members with chronic physical illness: A critical review of caregiver literature
Source: Health Qual Life Outcomes. 2004 Sep 17;2:50. doi: 10.1186/1477-7525-2-50 (PMC521496; doi:10.1186/1477-7525-2-50)
Supplement: Additional File 1 — Appendix 1: Review of empirical findings on quality of life of family caregivers. Author, topic, significant predictor variables, mediating variables, outcome variables, measurement, and intervention of 19 reviewed articles are shown in detail. [file 1477-7525-2-50-S1.doc]

**Additional File - Appendix 1: Review of empirical findings on quality of life of family caregivers**

| **Author** | **Topic** | **Significant**  **Predictor variables** | Mediating variables | **Outcome variables (Measurement)** | **Intervention** |
| --- | --- | --- | --- | --- | --- |
| Weitzner, &  McMillan [28] | Family caregiver QOL:  differences between  curative and palliative  cancer treatment settings | 1.Pt’s performance status  2.Educational status of  the caregiver |  | **Quality of life**  (the caregiver quality of life index-  cancer, 1997) | 1.Educating health care  professionals in both active  and palliative care settings |
| Haley et al.  [12] | Stress, appraisal, coping,  and social support as  predictors of adaptational  outcome among dementia caregivers | 1.Stressors (patient  impairment on the IADL)  2.Appraisal  3.Social support and activity  4.Coping responses | 1.Appraisal  2.Social support and  activity 3.Coping responses | **Adaptational outcome** 1.Depression (Beck Depression  Inventory, 1961)  2.Life satisfaction  (life satisfaction index from Z)  3.Health  (Self-rated health problems) | 1.Comprehensive intervention  with caregivers that includes  attention to caregiver cognition,  problem-solving, and social  support |
| Wallhagen [13] | Perceived control and  adaptation in elder  caregivers | 1.Subjective demands of  caregiving  2.Objective context  3.Subjective context  4.Perceived control | 1.Perceived control  2.Wishful thinking  coping behaviors | **Adaptation**  1.Caregiver’s level of life  satisfaction (the life satisfaction  index A, 1961)  2.Depression and subjective  symptoms of stress(The Hopkins symptom checklist, 1974) | 1.Focused interventions which  may be designed to facilitate  balancing demands and  resources |
| Nijboer et al.  [29] | Determinants of  caregiving experiences  and mental health of  partners of cancer  patients | 1.Caregiver characteristics  (income, quality of  relationship, initial  depression, initial  quality of life)  2.patient characteristics  (depression) | 1.Caregiver experience  (loss of physical  strength,  self-esteem) | Mental health outcome  1.Depression (20-item center for  epidemiologic studies depression  scale, 1991)  2.Quality of life (1-item linear  visual analogue self-assessment  scale, 1995) | 1.Health professionals’  involvement in the ongoing  care of cancer patients and  their families in order to be  aware of increasing demands |
| Jones, &  Peters [7] | Caring for elderly  dependents : effects  on the caregiver’s quality  of life | 1.patient characteristics  (age, dependency,  disability, depression)  2.carer characteristics  (relationship, age, sex,  disability)  3.effects on caregiver’s life  (social life, family life,  loneliness) |  | **Quality of life** 1.Stress  2.Anxiety  3.Depression  4.Health | 1.Support from community  services  2.Respite or relief from the caring  role |
| Boyle et al  [21] | Caregiver quality of  life after autologous bone  marrow transplantation | 1.demands of role change  2.responsibility  3.support |  | **Life satisfaction**  (8-item, open-ended questionnaire,  1993) | 1.Caregiving education in the  BMT experience |

| Clipp, &  George [30] | Dementia and cancer  : a comparison of spouse  caregivers | 1.The kind of illness |  | **Quality of life** 1.Physical health  2.Emotional health (the affect  balance scale, 1969)  3.Use of psychotropic drugs  4.Caregivers’ social life  5.Financial status (economic  resources section of the  OARS Methodology, 1988) |  |
| --- | --- | --- | --- | --- | --- |
| Miaskowski,  et al. [33] | Differences in patients’  and family caregivers’  perceptions of the pain  experience influence  patient and caregiver  outcomes | 1.Differences in the  perception of the pain  experience between  patients and their family  caregivers |  | **Quality of life**  (the Multidimensional Quality of Life  Scale-Cancer ; Padilla, 1992) | 1.Education on how to evaluate  the intensity of the patient’s pain  2.Education on effective ways to  communicate |
| Winslow [24] | Effects of formal supports  on stress outcomes in  family caregivers of  Alzheimer’s patients | 1.Care receiver dependency  2.Care receiver problem  behavior  3.Caregiver overload | 1.Coping  2.Formal support | **Stress outcomes** 1.Yielding of role  2.Physical health  3.Anxiety | 1.The expansion of funding  for long-term care  (health policy) |
| Cameron  et al. [22] | Lifestyle interference  and emotional  distress in family  caregivers of  advanced cancer  patients | 1.Education level  2.Caregiving assistance | 1.Lifestyle interference | **Emotional distress**  (the short form of the profile of  mood states, 1983)  1.Depression  2.Tension  3.Total mood disturbance | 1.Assisting caregivers  maintain participation in  activities  2.Providing caregivers with  greater preparation for their  role as caregivers |
| Goode et al.  [14] | Predicting longitudinal  changes in caregiver  physical and mental  health : a stress process  model | 1.Stressors | 1.Appraisals  2.Coping responses  3.Social support | 1.Caregiver mental health  (The center for epidemiological  studies depression scale ; 1977)  2.Caregiver physical health  (the Cornell medical index, 1986) | 1.Clinical intervention which  might focus on increasing the  relative use of approach  coping, modifying  stressfulness, and increasing  social support |
| Vitalino  et al. [26] | Predictors of burden  in spouse caregivers  of individuals with  Alzheimer’s disease | 1.Baseline burden  2.Care recipient ADLs  3.Caregiver vulnerability  4.Resources |  | **Caregiver distress** 1.Burden (the screen for caregiver  burden, 1991)  2.Depression (the beck depression  inventory, 1972)  3.Anxiety (the symptom checklist-  90 anxiety scale, 1977) | 1.Development of screening  protocols  2.Specific interventions to reduce  vulnerability and increase  resources |
| Pot, Deeg, & Dyck [23] | Psychological distress of  caregivers : moderator  effects of caregiver  resources? | Caregiving appraisal |  | **Caregivers’ Psychological**  **distress** (well-being)  (General health questionnaire,  1988) | 1.Need of attention and support  for caregivers who perceive  much pressure |

| Lowenstein [27] | The perception of  caregiving burden on the  part of elderly cancer  patients, spouses and  adult children | The nature of the family  relationship |  | **Caregiving burden**  1.Role strain  2.Personal strain  (Zarit burden scale, 1980) | 1.Provision of support  2.Activation of support group  3.Linking the patients to  community services  4.Advocacy on their behalf |
| --- | --- | --- | --- | --- | --- |
| Vedhara  et al. [25] | The role of stressors  and psychosocial  variables in the stress  process: a study of  chronic caregiver stress | Stressors | 1.Social support  2.Coping style | **Stress response**  1.Anxiety and depression  (savage personality screening  scale)  2.Stress (global measure of  perceived stress) |  |
| Schumacher  et al. [15] | The stress process in  family caregivers of  persons receiving  chemotherapy | 1.Caregiver age and gender  2.Patient age and gender  3.Patient functional status  4.The presence of recurrent  disease  5.Perceived efficacy of  coping strategies  6.Perceived adequacy of  social support | 1.Coping  2.Social support | 1.Depression (the profile of  mood states, 1971)  2.Strain (caregiver strain index,  1983) |  |
| Dunn et al.  [32] | Quality of life for  spouses of CAPD patients | 1.Marital adjustment  2.Income |  | **Quality of life**  (The quality of life index, 1985) | 1.Early intervention through  the use of marriage counseling  2.Education  3.Counseling with the social  worker  4.Referral to a social worker  about the financial burden  5.Spouse support group |
| Helder et al.  [31] | Living with Huntington’s  disease: Illness  perception, coping  mechanisms, and  spouses’ QOL | 1. Illness perceptions  2. Coping mechanisms |  | **Quality of life**  (The Medical Outcome Study 36-  item Short Form Health Survey,  1992) |  |
| Ergh et al.  [34] | Predictors of caregiver  and family functioning  following traumatic brain  injury: | 1. Neurobehavioral  disturbance  2. Caregiver perceived social  support | 1. Social support | **Caregiver distress**  (Brief Symptom Inventory, 1983) | 1.Seeking and obtaining  adequate social support |
